# 82. Comparative Evaluation of Low Versus High Doses of Rifampin for the Treatment of Staphylococcal Bone and Joint Infections: an open-label, randomized, controlled non-inferiority trial (EVRIOS)

**DOI:** 10.1093/ofid/ofae631.019

**Published:** 2025-01-29

**Authors:** Cedric Arvieux, Jean-Marc Chapplain, Anne Jolivet-Gougeon, Jean-François Morcet, Violaine Benoit, Cecile Lefeuvre, Florian Lemaitre, Nathalie Asseray, Aurélien Lorleac’h, Frederic Antoine Dauchy, Thomas Guimard, Gwenael Lemoal, Valérie Gaborieau, Grégory Corvaisier, Rodolphe Buzele, Pierre Delobel, Fabien Fily, Pierre Abgueguen, Jocelyn Michon, Florence Suy, Anne Méheut, marie Gheno, Maja Ratajczak, Anaïs Grèves, Céline Thomas, Mustapha Ahmim, Romain Muraz, Bellec Laurent, Cailleaux Marine, Lecomte Raphael, Séverine Ansart, Louis Bernard

**Affiliations:** CHU de Rennes, Rennes, Bretagne, France; CHU de Rennes, Rennes, Bretagne, France; CHU RENNES, RENNES, Bretagne, France; CHU RENNES, RENNES, Bretagne, France; CHU RENNES, RENNES, Bretagne, France; CHU RENNES, RENNES, Bretagne, France; CHU RENNES, RENNES, Bretagne, France; CHU DE NANTES - HOTEL DIEU, NANTES, Pays de la Loire, France; CH BRETAGNE SUD, LORIENT, Bretagne, France; CHU BORDEAUX, BORDEAUX, Aquitaine, France; CHD VENDEE, LA ROCHE / YON, Pays de la Loire, France; CHU POITIERS, POITIERS, Poitou-Charentes, France; CH PAU, PAU, Aquitaine, France; CH VANNES, VANNES, Bretagne, France; CH SAINT-BRIEUC, SAINT-BRIEUC, Bretagne, France; CHU TOULOUSE, TOULOUSE, Midi-Pyrenees, France; CH SAINT-MALO, SAINT-MALO, Bretagne, France; CHU ANGERS, ANGERS, Pays de la Loire, France; CHU DE CAEN, CAEN, Basse-Normandie, France; Hôpital Privé Jean Mermoz - Clinique du Parc / LYON, LYON, Rhone-Alpes, France; CHU RENNES, RENNES, Bretagne, France; CHU DE RENNES, RENNES, Bretagne, France; CHU de Rennes, Rennes, Bretagne, France; CHU BREST, BREST, Bretagne, France; CHU POITIERS, POITIERS, Poitou-Charentes, France; CHU DE RENNES, RENNES, Bretagne, France; CHU DE RENNES, RENNES, Bretagne, France; CH de Pontivy, Pontivy, Bretagne, France; CHU de Rennes, Rennes, Bretagne, France; CHU de Nantes, Nantes, Pays de la Loire, France; CHU DE BREST, BREST, Bretagne, France; CHU TOURS, TOURS, Centre, France

## Abstract

**Background:**

The antibiotic management of staphylococcal bone and joint infection usually relies on rifampin associated with another antibiotic for susceptible strains. The appropriate dose of rifampin remains unclear and diverges in international recommendations.

**Figure:**
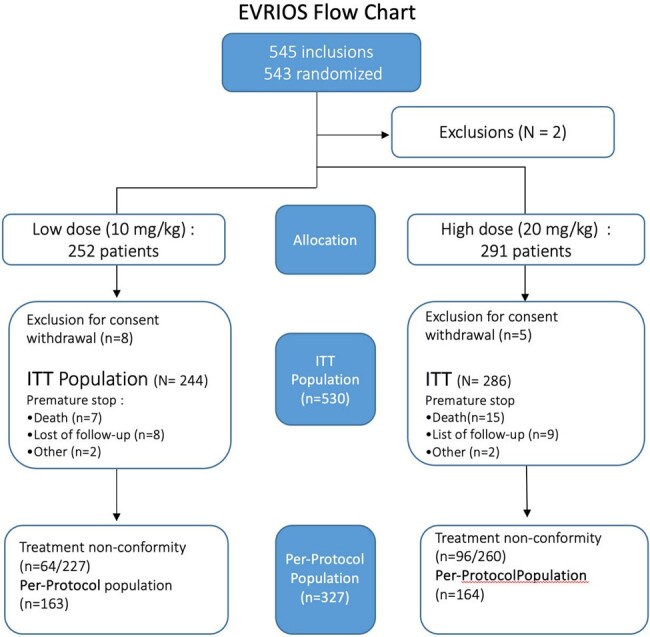

543 patients were randomized, 530 were included in the Intent-to-Treat analysis and 327 in the per-protocol analysis.

**Methods:**

We performed an open-label, randomized, controlled, noninferiority trial to compare a daily dose of 10 mg/kg (Low-dose) qd and 20 mg/kg (High-dose, divided in 10mg/kg bid for high BMIs) of rifampin in association with another antibiotic in patients with microbiologically confirmed bone and joint infections due to *Staphylococcus sp*. Surgical procedures were performed as needed. The primary outcome was persistent infection, defined as the persistence or recurrence of infection with the initial causative bacteria within 1 year after the completion of antibiotic therapy.

**Results:**

A total of 543 patients from 18 French centers were randomly assigned to receive either Low-dose (252 patients) or High-dose (291 patients) of rifampin associated with another antibiotic. Thirteen patients who withdrew consent were not included in the analysis. The main antibiotics associated with rifampin were levofloxacin/ofloxacin (82%, 435 out of 530 patients). Mean age was 59.8 ± 16.4 years, and mean BMI 27.9 ± 5.8 kg/m^2^; 24.6% [n=130] had osteosynthesis and 34% [n=180] prostheses at the site of infection. *Staphylococcus aureus* was present in 75.8% of patients [n=402].

In the ITT analysis, persistent infection occurred in 8 of 227 patients (3.5%) in the Low-dose group and in 10 of 265 patients (3,8%) in the High-dose group (risk difference, 0.0019; 95% confidence interval [-0.032 – 0.036], thus, noninferiority was shown. Serious adverse events linked to rifampin where more frequent in the High-dose group (7%) than in the Low-dose group (1.6%, p=0.0043). Patients in the High-dose group were less likely to remain in the per-protocol analysis (57%, versus 67% in the Low-dose group). Noninferiority was also shown in the per-protocol analysis.

**Conclusion:**

Among patients with confirmed *Staphylococcus sp*. bone and joint infection that were managed with standard surgical procedures, antibiotic therapy with daily 10 mg/kg of rifampin was shown to be noninferior to 20 mg/kg. Severe adverse events linked to rifampin where more frequent in the 20 mg/kg group.

**Disclosures:**

**Cedric Arvieux, MD**, GILEAD: Advisor/Consultant|ViiV: Advisor/Consultant **Florian Lemaitre, PharmD**, Astellas: Grant/Research Support|Chiesi: Grant/Research Support|Pfizer: Scientific meeting fees|ViiV: Scientific meeting fees **Aurélien Lorleac'h, MD**, Gilead: Hospitality|GSK: Hospitality|Nestlé Home Care: Hospitality|ViiV Healthcare: Hospitality **Thomas Guimard, MD**, Gilead: Convention invitation|Menarini France: Hospitality|MSD: Hospitality|Pfizer: Advisor/Consultant|Shionogi BV: Hospitality **Gwenael Lemoal, MD**, Gilead: Hospitality|MSD: Hospitality|Viiv Heathcare: Advisor/Consultant

